# The response of ducks to V4 Newcastle disease virus and its transmission to contact ducks and domestic chickens

**Published:** 2014

**Authors:** Majid Bouzari

**Affiliations:** Department of Biology, Faculty of Sciences, University of Isfahan, Isfahan, Iran.

**Keywords:** Domestic chicken, Duck, Newcastle disease virus strain V4, Transmission

## Abstract

Experimental infection of Muscovy ducks with V4 strain of Newcastle disease virus was undertaken to determine the response of the ducks to the virus and the possibility of virus transmission to ducks and chickens in village like conditions. Twelve ducks were randomly and equally divided into three groups of control, inoculated and in-contact. Additionally, the chickens were placed into two groups of four animals each, namely in-contact and control. The inoculated and in-contact ducks and in-contact chickens were kept together. The eye drop route was used for inoculation and hemagglutination inhibition (HI) antibodies were measured for assessment of antibody response and cloacal and pharyngeal swabs were used for detection of the virus. The primary antibody response of inoculated ducks was very high and rapid (geometric mean titers [Log base 2] of up to 5.75 ± 0.50). The in-contact ducks showed antibody response with the same pattern but lower titers than the inoculated ducks (geometric mean titers [Log base 2] of up to 3.25 ± 1.70). The in-contact chickens showed a slight increase of HI antibody (geometric mean titers [Log base 2] of up to 2.25 ± 1.25) while the control chickens did not show any increase. The antibody response indicated the transmission of the virus to contact ducks and chickens. A single isolation of virus confirmed the ability of ducks to excrete the virus. It was concluded that the V4 strain of Newcastle disease virus was highly antigenic for ducks, and ducks can transmit it to other ducks and also in-contact chickens.

## Introduction

Newcastle Disease Virus (NDV) occurs worldwide and infects different kinds of birds including chickens. The disease can be fatal for them. By serological tests and virus detection it has been shown that ducks can be infected by NDV.^1^^-^^13^ There are few reports of death of affected ducks^6^^,^^13^ and ducklings,^5^ and susceptibility of these birds is much lower than those of fowls.^12^^-^^15^ In different surveys regarding ducks infection, both mesogenic^1^ and velogenic strains ^7^^,^^11^^,^^16^ have been detected, but most of the isolated strains were lentogenic.^8^^-^^10^^,^^12^^,^^17^^,^^18^

Serologically, it has been shown that experimentally infected ducks with velogenic NDV could transmit virus to in-contact free-range chickens.^19^ Sudharma and Sulochana have recorded that chickens in-contact with ducklings infected with Herts strain of NDV contracted the infection.^5^ According to Majiyagbe and Nawathe the velogenic NDV pathotype recognized in Nigeria has also been isolated from domestic ducks that have been mixed with the local breed of chicken.^7^ The isolation of the same virus strain from apparently normal domestic ducks can lead to speculations that ducks may be a source of infection to local breeds of the chickens.

While the most strains isolated from ducks have been lentogenic, the studies on the transmission of NDV from ducks to chickens has been associated mostly with velogenic strains, hence the role of ducks in dissemination of lentogenic strains has remained unclear. The aim of this study was to test the response of ducks to V4 virus (a lentogenic strain of NDV) using antibody response, virus isolation and transmission of virus to in-contact ducks and chickens.

## Materials and Methods

**Ducks and chickens. **Ducks were obtained from a small flock kept at a private farm in Brisbane, Australia. The ducks were young adults and the females came into lay during the experiment. They were randomly divided into three groups: control, inoculated and in-contact groups (four in each). The breeds of the ducks in each group are shown in Table 1. The chickens used were eight young domestic chickens divided into in-contact and control groups. Inoculated ducks, together with in-contact chickens and ducks were kept in the same place while the control groups were placed 260 meters from experimental groups. Before commencing the trial, blood samples were collected from all birds for detecting pre-existing antibody against NDV.

**Table 1:** The breeds of experimental ducks in different groups

**Group**	**Label Number**	**Breed**
**Control ducks**	D1	White Muscovy
	D2	White Muscovy
	D3	Blue Pied Muscovy
	D4	White Muscovy
**Contact ducks**	D5 White Campbell (Indian Runner)	
	D6	Black Pied Muscovy
	D7	White Muscovy
	D8	White Muscovy
**Inoculated ducks**	D9	Blue Pied Muscovy
	D10	Blue Pied Muscovy
	D11	White Muscovy
	D12	White Muscovy

**Virus and inoculation. **The inoculum used was suspension of NDV-V4 in phosphate-buffered saline (PBS) with a titer of 10^7^^.^^5^ EID 50 per 0.1 mL (50% chicken embryo infective dose). The titer was calculated by the technique of Reed and Muench.^20^ Strain V4 is a lentogenic strain of NDV which has been used as vaccine in chickens. The intraocular route was used for inoculation.

**Serological test and studies on antibody response. **Blood was collected through brachial vein a week before inoculation and for three consecutive weeks after virus inoculation to study antibody responses. After the collection, blood was allowed to clot and kept in hot room (37 ˚C) for 3 hr. The serum was decanted and then frozen at –20 ˚C until tested. In order to remove natural agglutinins all the sera were treated with chicken red blood cells (RBC). For this purpose, a 10% suspension of RBC in dextrose-veronal-glucose (DVG) was centrifuged 3 min at 250 *g*. The fluid was sucked off, then the cells were suspended and one drop of the suspended cells were added to each serum sample by a Pasteur pipette, mixed and kept in refrigerator (4 ˚C) for 20 min and then centrifuged at 250 *g* for 15 sec. The serum was decanted and then tested using a micro hemagglutination inhibition (HI) test.^21^ Serial two-fold dilution of sera were reacted with 4 hemagglutination (HA) units of NDV-V4 for 20 min at room temperature. Chicken RBC (1% suspension) were added and the test was read after an additional 45 min. The last dilution with complete inhibition of HA was recorded as HI titer in the serum.

**Studies on transmission. **The ducks in test group were exposed to the lentogenic strain of NDV-V4 by eye drop. A quantity of 0.1 mL of the virus suspension was administered into the eye. The frequency of sampling was scheduled to obtain information on the presence and transmission of the virus, and on the development of antibodies. Pharyngeal and cloacal swabs were taken from all birds daily for 14 post-inoculation days and then weekly for 3 weeks. The swabs were placed in ampoules containing 1 mL PSG (penicillin 10.74 g, streptomycin 500 mg, gentamicin 250 mg, PBS 100 mL /make up to 1 liter with distilled deionized water plus 5% calf serum) and frozen at –70 ˚C until tested. To attempt virus isolation, swabs for the first 14 days were tested individually. Then, samples were pooled, with four birds from each group being amalgamated on each collection day. A volume of 0.20 mL of transport medium was inoculated into each of three 10-day-old embryonated chicken eggs. Eggs were incubated at 37 ˚C. After 72 hr of incubation, they were chilled, opened and the harvested allantoic fluids were tested in a standard HA test.^22 ^Serial two-fold dilutions of sera were used and geometric mean titers were expressed as log index to base 2. 

## Results

**Clinical response. **No specific clinical signs were observed in the ducks and chickens. In the second week of the experiment, only one duck showed weakness and died after one week. In histopathologic tests, the cause of death was associated with lymphoproliferative disease. No bacteria were isolated.

**Fig. 1:**
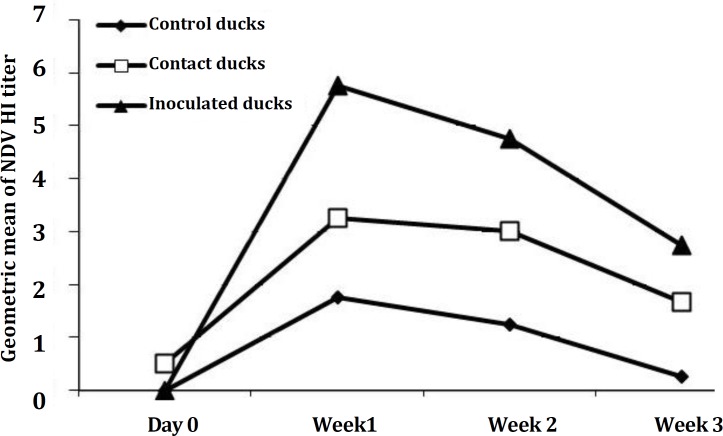
Geometric mean of NDV HI titer (Log base 2) of inoculated, in-contact and control ducks

**Fig. 2:**
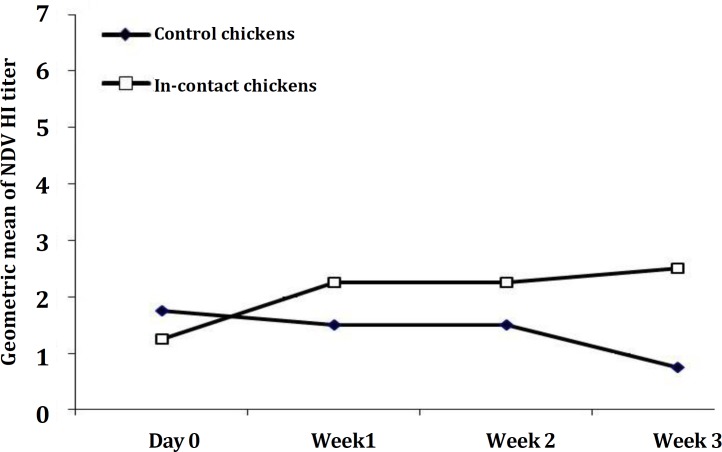
Geometric mean of NDV HI titer (Log base 2) of in-contact and control chickens

**Serological response of the different experimental groups. **The mean of antibody titers of different groups are shown in Table 2 and Figures 1 and 2. As it is shown in Figure 1, the geometric mean titers of the three groups were all under 1 at the start of the study. The vaccinated ducks appeared to have a response with geometric titers peaking at about 6 after one week. The contacts also developed antibodies rapidly, within one week on initiation of contact. Geometric mean titers for this group were generally lower than those of the vaccinated group. The chickens had low levels of antibody at the start of the experiment. Those in contact with the inoculated ducks showed an increase in titer during the experiment, to geometric means in excess of 2.5. The levels of antibody in control chickens declined to negligible levels (Fig. 2).

**Isolation of virus from swabs. **The virus was only isolated once from the pharynx of one of the ducks in the inoculated group, 3 days after exposure.

**Table 2:** Geometric mean of NDV (Log base 2) of HI antibody titers (± standard deviation) in all groups of ducks and chickens (* n = 3).

**Time **	**No.**	**Ducks**				**Chickens**	
		**Control**	**In-contact**	**Inoculated**		**Control**	**In-contact**
**Day 0**	**4**	0	0.50 ± 1.00	0		1.75 ± 0.50	1.25 ± 1.25
**Week ** **1**	**4**	1.75 ± 1.50	3.25 ± 1.70	5.75 ± 0.50		1.50 ± 1.29	2.25 ± 1.25
**Week ** **2**	**4**	1.25 ± 1.25	3.00 ± 1.82	4.75 ± 0.50		1.50 ± 0.57	2.25 ± 0.50
**Week ** **3**	**4**	0.25 ± 0.50	1.66 ± 0.57*	2.75 ± 0.50		0.75 ± 0.50	2.50 ± 0.57

## Discussion

The V4 strain of Newcastle disease virus infects chickens by different routes, spreads rapidly between chickens and is heat resistant.^23^^,^^24^ It has been used as vaccine in different parts of Asia. There have been many studies in assessing the antibody response of chickens to V4, but has not been any previous experience with the response of ducks to this virus. 

There are reports about the potential dissemination of NDV by ducks, based on serological and also isolation of lentogenic, mesogenic and velogenic strains of the virus from domestic and feral ducks.^1^^-^^13^^,^^18^^, ^^25^ There is little information on the possibility of transmission of the lentogenic V4 strain to other ducks and chickens. Thus, this experiment tried to assess the response of ducks to V4 strain of NDV and also the possibility of its transmission via direct contact to ducks and chickens.

There is no basic data on the levels of expected HI antibodies in ducks. Perusal of the pre-vaccination titers suggests that titer levels of up to 2 may indicate non-specific reactions in duck serum. The responses to vaccination supported this. Vaccine titers were consistently over 2, and individual titers of above 6 were recorded.

In contrast to one report showing failure of immune response in ducks exposed to NDV,^13^ the inoculated ducks in this experiment had a very high antibody response after one week. This supports other reports suggesting that NDV is highly antigenic for ducks.^6^^,^^18^ The HI antibody titers were much higher in ducks than chickens in the present experiment, and higher than the titers usually recorded in chickens. The immune response in the in-contact ducks indicates the transmission of the virus between ducks. The results could have some application when this strain of the virus is considered for vaccination especially in village conditions.

In-contact chickens there was slight increase in HI antibody that indicated they had also been infected by the virus, but the amount of antibody in the first week was not protective. According to Allan and Gouph the HI titers of 8 (i.e. 2^3^) are protective.^26^ Potentially protective levels of antibody were not achieved by the third week. This modest response of chickens to primary exposure to the V4 virus was similar to previous studies with the V4 strain of NDV.^23^ In contrast, the control chickens gave no serological evidence of contact with NDV.

According to this study, it is suggested that in village conditions where the ducks and chickens are kept together or their chance of contact is highly possible, simultaneous vaccination of chickens and ducks is advisable. 
